# Prognostic Value of Pulmonary Transit Time and Pulmonary Blood Volume Estimation Using Myocardial Perfusion CMR

**DOI:** 10.1016/j.jcmg.2021.03.029

**Published:** 2021-11

**Authors:** Andreas Seraphim, Kristopher D. Knott, Katia Menacho, Joao B. Augusto, Rhodri Davies, Iain Pierce, George Joy, Anish N. Bhuva, Hui Xue, Thomas A. Treibel, Jackie A. Cooper, Steffen E. Petersen, Marianna Fontana, Alun D. Hughes, James C. Moon, Charlotte Manisty, Peter Kellman

**Affiliations:** a Institute of Cardiovascular Science, University College London, Gower Street, London, United Kingdom; b Barts Heart Centre, St Bartholomew’s Hospital, West Smithfield, London, United Kingdom; c National Heart, Lung, and Blood Institute, National Institutes of Health, DHHS, Bethesda, Maryland, USA; d William Harvey Research Institute, Queen Mary University of London, United Kingdom; e Royal Free Hospital, Pond Street, London, United Kingdom

**Keywords:** first pass perfusion, outcomes, pulmonary blood volume, AIF, arterial input function, CI, confidence interval, ICD, implantable cardioverter-defibrillator, IQR, interquartile range, MBF, myocardial blood flow, MPR, myocardial perfusion reserve, PBV, pulmonary blood volume, PTT, pulmonary transit time, PTTn, pulmonary transit time normalized for heart rate

## Abstract

**Objectives:**

The purpose of this study was to explore the prognostic significance of PTT and PBVi using an automated, inline method of estimation using CMR.

**Background:**

Pulmonary transit time (PTT) and pulmonary blood volume index (PBVi) (the product of PTT and cardiac index), are quantitative biomarkers of cardiopulmonary status. The development of cardiovascular magnetic resonance (CMR) quantitative perfusion mapping permits their automated derivation, facilitating clinical adoption.

**Methods:**

In this retrospective 2-center study of patients referred for clinical myocardial perfusion assessment using CMR, analysis of right and left ventricular cavity arterial input function curves from first pass perfusion was performed automatically (incorporating artificial intelligence techniques), allowing estimation of PTT and subsequent derivation of PBVi. Association with major adverse cardiovascular events (MACE) and all-cause mortality were evaluated using Cox proportional hazard models, after adjusting for comorbidities and CMR parameters.

**Results:**

A total of 985 patients (67% men, median age 62 years [interquartile range (IQR): 52 to 71 years]) were included, with median left ventricular ejection fraction (LVEF) of 62% (IQR: 54% to 69%). PTT increased with age, male sex, atrial fibrillation, and left atrial area, and reduced with LVEF, heart rate, diabetes, and hypertension (model r^2^ = 0.57). Over a median follow-up period of 28.6 months (IQR: 22.6 to 35.7 months), MACE occurred in 61 (6.2%) patients. After adjusting for prognostic factors, both PTT and PBVi independently predicted MACE, but not all-cause mortality. There was no association between cardiac index and MACE. For every 1 × SD (2.39-s) increase in PTT, the adjusted hazard ratio for MACE was 1.43 (95% confidence interval [CI]: 1.10 to 1.85; p = 0.007). The adjusted hazard ratio for 1 × SD (118 ml/m^2^) increase in PBVi was 1.42 (95% CI: 1.13 to 1.78; p = 0.002).

**Conclusions:**

Pulmonary transit time (and its derived parameter pulmonary blood volume index), measured automatically without user interaction as part of CMR perfusion mapping, independently predicted adverse cardiovascular outcomes. These biomarkers may offer additional insights into cardiopulmonary function beyond conventional predictors including ejection fraction.

The pulmonary circulation is inextricably linked with cardiac physiology, but our understanding of the cardiopulmonary axis in various disease states is limited. Use of noninvasive imaging biomarkers as surrogate indicators of cardiopulmonary status may facilitate risk stratification and outcome prediction, potentially contributing to personalized clinical care.

Pulmonary transit time (PTT) and pulmonary blood volume (PBV) are physiological parameters reflective of cardiopulmonary hemodynamics (1). Both are known to be altered in various disease states, including heart failure (2,3), pulmonary hypertension (4,5), and chronic lung disease (6), and to correlate with structural, functional, and biochemical parameters of pulmonary (7) and cardiac function (8). Pulmonary transit time, defined as the time interval for a contrast bolus to pass from the right- to left-sided circulation, and PBV (the product of PTT and cardiac output), correlate with established prognostic biomarkers, including right ventricular (RV) and left ventricular (LV) ejection fraction (9), markers of LV diastolic function (10), brain natriuretic peptide levels (9), and pulmonary vascular resistance (4). Importantly, a small number of studies suggested an independent prognostic utility of PTT and PBV in specific disease models (2,5,11).

Despite extensive research supporting a clinical utility of PTT and PBV, at-scale analysis and clinical adoption have been hindered by challenges in data acquisition, requiring either invasive catheterization (1) or manual segmentation and data extraction from noninvasive tests (2,5, 6, 7, 8, 9, 10). Recent developments in quantitative cardiovascular magnetic resonance (CMR) perfusion permit automated estimation of PTT inline as part of routine perfusion mapping without the need for additional acquisitions or processing, enabling large data analyses and potentially facilitating clinical adoption.

In this study, a fully automated machine learning approach for identification of RV and LV arterial input functions during first-pass perfusion imaging was deployed, allowing in-line estimation of PTT and subsequent calculation of PBV. Using a large, 2-center patient cohort, we investigated the potential clinical utility of PTT and PBV by assessing correlations with other parameters and any independent prognostic significance.

## Methods

### Patients and study design

This was a retrospective cohort study of patients referred for adenosine stress CMR at 2 centers (Barts Heart Center and the Royal Free Hospital, London, United Kingdom), between March 2016 and August 2018. This cohort has been used to explore the prognostic effect of myocardial blood flow (MBF) and myocardial perfusion reserve (MPR), and has been previously described (12). In brief, consecutive adult patients referred for a myocardial perfusion scan were included. Patients with congenital heart disease, known intracardiac shunts (known to affect methods based on the indicator dilution principles [13]), and inherited or infiltrative cardiomyopathies (hypertrophic cardiomyopathy and cardiac amyloid) were excluded.

The primary outcome was the incidence of major adverse nonfatal cardiovascular events (defined as myocardial infarction, stroke, heart failure admission, and ventricular tachycardia or appropriate implantable cardioverter-defibrillator [ICD] treatment [including ICD shock and/or antitachycardia pacing]). All-cause mortality was defined as a secondary outcome in view of the sample size and the broadly unselected patient population, which meant that the cause of death was often unrelated to cardiorespiratory disease and included sepsis and cancer-related deaths. Mortality data was obtained from the National Health Service Spine portal, with data on the cause of death being available for only a small number of patients. Clinical data were retrieved from the electronic patient records, with follow-up starting from the date of the perfusion CMR examination. Outcomes for the primary analysis were censored at death and end of follow-up period. Comorbidities recorded included history of hypertension, dyslipidemia, diabetes mellitus, atrial fibrillation, stroke or transient ischemic attack, smoking, history of previous myocardial infarction, percutaneous coronary intervention (PCI) or coronary artery bypass surgery (CABG), and cancer (all based on medical records).

The study was approved by the National Health Service Research Ethics Committee and Health Research Authority (Barts BioResource with permission from REC ID 14/EE/0007, Royal Free Hospital: REC ID 07/H0715/101). The study conformed to the principles of the Helsinki Declaration, and all patients provided written, informed consent.

### Cardiovascular magnetic resonance

CMR studies were carried out on 1 of 4 1.5-T (Aera) or 3-T scanners (Prisma, Siemens Healthineers, Erlangen, Germany). A standard clinical protocol, including cine imaging and stress and rest perfusion followed by late gadolinium enhancement, was used for all studies. Stress myocardial perfusion was performed, using adenosine as pharmacological stressor according to guidelines (14). The myocardial perfusion sequence is a single-bolus, dual sequence described previously (15). Basal, midventricular, and apical short-axis perfusion images were acquired at both stress and rest. Image acquisition was performed over 60 to 90 heartbeats and a bolus of 0.05 mmol/kg gadoterate meglumine (Dotarem, Guerbet, Paris, France) was administered at 4 ml/s during both maximal hyperemia and subsequently at rest (for estimation of stress and rest MBF respectively). MPR was defined as the ratio of stress MBF over rest MBF. PTT data was calculated from perfusion imaging, and PBV was estimated utilizing resting cardiac output measurement from cardiac volumes obtained from short-axis stack cine images.

### Imaging analysis

Image analysis for cardiac volume parameters and presence and distribution of late gadolinium parameters was performed using commercially available software (CVI42, Circle Cardiovascular Imaging, Calgary, Alberta, Canada). The perfusion sequence deployed (15) involves the simultaneous acquisition of separately optimized sequences for myocardium and blood pool signals. Motion-corrected low-resolution dynamic images from a basal short-axis view are used to extract the arterial input function (AIF) of the RV and LV. The quantitative mapping uses a convolutional neural network approach to automatically segment the LV and RV cavities, thereby allowing the estimation of arterial input function (signal intensity over time) for both ventricles during first pass of contrast. The blood pool detection process was described in detail previously (16). The resulting signal intensity curves are then converted to gadolinium concentration (mmol/l) based on automatically generated look-up tables for the magnetization Bloch simulation. Reconstruction and post-processing are executed within the Gadgetron software framework (17), allowing in-line estimation of the time interval between the RV and LV curve AIF curves.

### PTT and PBV estimation

**Figure 1:**
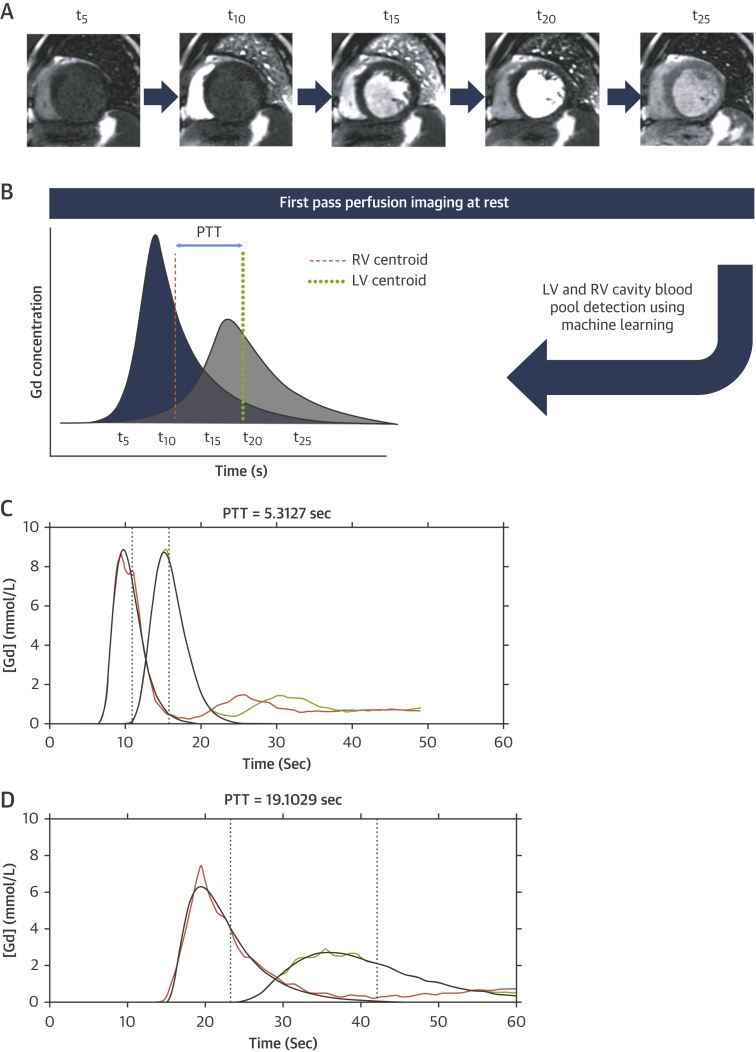
Automated, Inline Method of Pulmonary Transit Estimation **(A)** Dynamic first-pass perfusion imaging of a basal short-axis slice showing the right ventricular (RV) and left ventricular (LV) cavities (here high-resolution images). **(B)** Schematic gadolinium (Gd) time-concentration curves in the RV and LV cavities with the recirculation component removed for clarity. The **dashed lines** indicate the location of the centroid in each cavity, and the difference (i.e., the pulmonary transit time [PTT]) between each centroid is indicated by the **arrow**. **(C and D)** Examples of rest PTT estimation in study patients. **(C)** A 59-year-old man, left ventricular ejection fraction (LVEF) = 72%, PTT = 5.3 s, pulmonary blood volume index (PBVi) 374 ml/m^2^. **(D)** A 57-year-old man, LVEF = 19%, PTT = 19.1s, PBVi 596 ml/m^2^

Non-invasive methods of volume estimation are based on the indicator dilution principle and have been previously validated against invasive thermodilution methods (18). The PTT was estimated as the time between the centers of gravity (centroids) of the RV and LV arterial input function curves, after exclusion of the recirculation component (Figure 1). The use of centroids of the AIF curves was previously shown to be superior to peak-to-peak methods for PBV estimation (19). Pulmonary transit time normalized for heart rate (PTTn) was estimated by dividing PTT with the duration of the cardiac cycle (R-R interval, in seconds) as performed in previous studies (8,9):

(eq 1) PTTn=PTT(s)/R-R interval(s)

Pulmonary blood volume was estimated as the product of PTT and cardiac output as originally described from indicator dilution methods (20):

(eq. 2) PBV=PTT×cardiac output

This was indexed to body surface area (BSA), allowing calculation of pulmonary blood volume index (PBVi):

(eq. 3) PBVi=PTT × cardiac output/BSA

The LV stroke volume was estimated using steady state free precession (SSFP) cine images from manual planimetry of a full short-axis stack in end-diastole and -systole, and the patient’s heart rate at rest was used to derive cardiac output (cardiac output = stroke volume × heart rate). Rest PTT was used for the primary analysis, including estimation of PBV as cardiac output data was only available during rest. Associations between stress PTT and outcomes were performed as a secondary exploratory analysis.

### Statistical analysis

Continuous variables were reported as mean ± SD when normally distributed and as median (interquartile range [IQR]) when not. Normality was assessed by visual inspection of the frequency histograms and quantified using a Kolmogorov-Smirnov test. Categorical variables were summarized as frequencies and percentages. Comparisons between MACE and non-MACE groups were performed for continuous variables using a 2-tailed unpaired Student’s *t-*test or a Mann-Whitney *U* test depending on normality, and categorical variables were compared with a chi-square test. Correlations were assessed using Spearman’s rank correlation coefficient. Predictors of rest PTT were evaluated using a multivariate regression analysis, the model of which included parameters either known to correlate with rest PTT as shown in previous studies (8), or that would have a physiological basis for interacting with PTT. Rest PTT was log-transformed in the regression model to meet the model assumptions. Unstandardized beta coefficients were obtained allowing predictors to be expressed in their original units. To identify independent prognosticators of MACE and all-cause mortality, separate Cox proportional hazard regression analyses were performed with adjustment for covariates including age, sex, left ventricular ejection fraction (LVEF), presence of late gadolinium enhancement (LGE), MPR, LA area index, diabetes mellitus, dyslipidemia, hypertension, and previous history of infarction/PCI/CABG. The proportional hazards assumption was checked using Schoenfeld residuals (Supplemental Figure S1). A sensitivity analysis was also performed to obtain Firth’s bias-adjusted estimates to ensure that there was no bias in the estimated coefficients due to the relatively low event rates. Results were similar to the original models. Survival curves were constructed according to the Kaplan-Meier method and compared dichotomous groups using the mean values for PTT and PBVi within the population as cut offs. A p value <0.05 was considered significant. Analysis was performed using SPSS Statistics, version 26.0 (IBM, Armonk, New York).

## Results

### Cohort description and baseline characteristics

A total of 1,049 patients with CMR myocardial perfusion imaging data were available for inclusion as previously described (12). Of these, 4 (0.4%) had confirmed intracardiac shunts and were therefore excluded, in addition to 60 (5.7%) patients with incomplete or erroneous rest perfusion data (including incorrect automated blood pool identification, incorrect timing of contrast administration, and poor AIF signal of either the RV or LV). A total of 985 patients with available rest PTT data were therefore included in the main analysis.

Median age of the patients was 62 years (IQR: 52 to 71 years) and 660 (67%) were men. There were 281 (28.6%) patients with diabetes mellitus, and 306 (31%) patients had a prior history of either PCI or CABG. The median LVEF across the cohort was 62% (IQR: 54% to 69%). Baseline characteristics are summarized in Table 1.

**Table 1:** Baseline Demographics and CMR Parameters of the Patient Cohort (N = 985)

Demographics	
Age, yrs	62 (52–71)
Male	660 (67)
Body surface area, kg/m^2^	1.90 (1.8–2.1)
Comorbidities	
Diabetes mellitus	281 (28.6)
Hypertension	590 (60)
Dyslipidemia	479 (48.7)
Atrial fibrillation	129 (13.1)
Previous stroke or TIA	58 (5.9)
Previous MI/PCI/CABG	306 (31)
Smoking history (current or previous)	337 (34.2)
Cancer (active or previous diagnosis)	100 (10.2)
CMR parameters	
LVEDVi, ml/m^2^	75 (64–91)
LVSVi, ml/m^2^	46 (40–53)
LVEF, %	62 (54–69)
LVMi, g/m^2^	57 (48–68)
LA area index, cm^2^/m^2^	11.8 (10.1–13.9)
Presence of LGE, n %	416 (42)
Stress MBF ml/g/min	1.98 (1.6–2.5)
Rest MBF, ml/g/min	0.89 (0.8–1.1)
MPR	2.39 (1.9–3.0)
Resting heart rate,^a^ beats/min	68 (61–77)
Cardiac output, l/min	5.97 (5.1–7.2)

Values are median (interquartile range) or n (%).

CABG = coronary artery bypass graft surgery; CMR = cardiac magnetic resonance; LA = left atrium; LGE = late gadolinium enhancement; LVEDVi = left ventricular end-diastolic volume index; LVEF = left ventricular ejection fraction; LVMi = left ventricular mass index; LVSVi = left ventricular stroke volume index; MBF = myocardial blood flow; MI = myocardial infarction; MPR = myocardial perfusion reserve; PCI = percutaneous coronary intervention; TIA = transient ischemic attack.

a At the time of rest perfusion acquisition.

### Analysis of associations among rest PTT, cardiac parameters, and clinical characteristics

**Figure 2:**
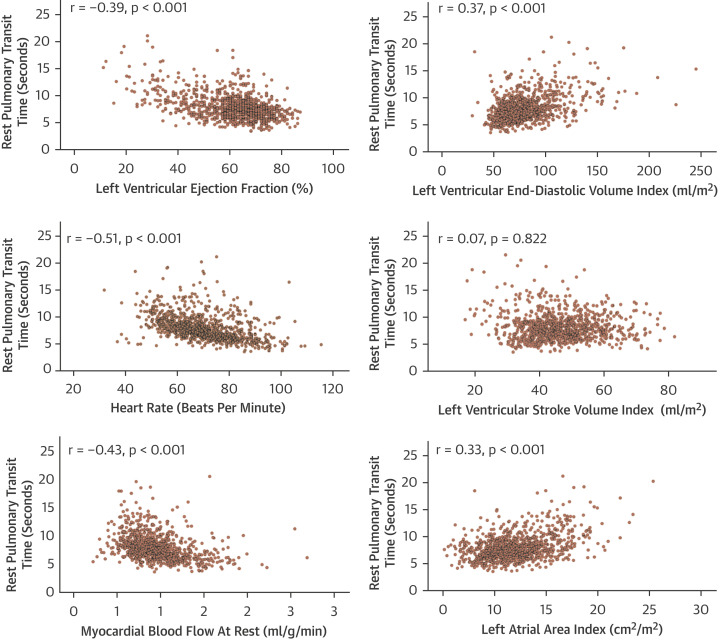
Associations of Rest PTT With Cardiac Parameters Spearman’s (rho) correlation of pulmonary transit time (PTT) with heart rate, cardiac volume parameters, left ventricular ejection fraction, left atrial area, and rest myocardial blood flow.

Median rest PTT was 7.7 s (IQR: 6.4 to 9.2 s). The median PBV index was 400 ml/m^2^ (IQR: 335 to 475 ml/m^2^). Rest PTT correlated with LV end-diastolic volume index (r = 0.37) and left atrial area index (r = 0.33), and negatively correlated with LVEF (r = −0.39), heart rate (r = −0.51), and myocardial blood flow at rest (r = −0.43) (Figure 2). In a multivariable regression analysis, LVEF (β = −0.007; 95% confidence interval [CI]: −0.008 to −0.006; p < 0.001), heart rate measured during rest perfusion (β = −0.008; p < 0.001), age (β = 0.003; p < 0.001), LA area index (β = 0.019; p < 0.001), atrial fibrillation (β = 0.118; p < 0.001), male (β = 0.052; p < 0.001), diabetes (β = −0.060; p < 0.001), hypertension (β = −0.038; p = 0.003), and rest myocardial blood flow (β = −0.098; p = 0.006) were independently associated with log_e_ PTT (Table 2). These predictors explained 57.0% of the variance in rest PTT.

**Table 2:** Relationship Between Rest PTT and Demographic and CMR Parameters: Multivariate Regression Analysis of Parameters Independently Associated With Log_e_ PTT

Independent Variables	Standardized Beta	β (Unstandardized)	95% CI of β	p Value
LA area index, ml/m^2^	0.21	0.019	0.015 to 0.023	<0.001
Heart rate, beats/ min	−0.38	−0.008	−0.010 to −0.007	<0.001
Age, yrs	0.14	0.003	0.002 to 0.004	<0.001
Atrial fibrillation	0.15	0.118	0.083 to 0.154	<0.001
LVEF, %	−0.35	−0.007	−0.008 to −0.006	<0.001
Diabetes mellitus	−0.10	−0.060	−0.087 to −0.034	<0.001
Hypertension	−0.07	−0.038	−0.064 to −0.013	0.003
Rest myocardial blood flow, ml/g/min	−0.11	−0.098	−0.146 to −0.050	<0.001
Male	0.09	0.052	0.026 to 0.078	<0.001

β = unstandardized beta; CI = confidence interval; PTT = pulmonary transit time; other abbreviations as in Table 1.

### Association of rest PTT and PBV with outcomes

**Figure 3:**
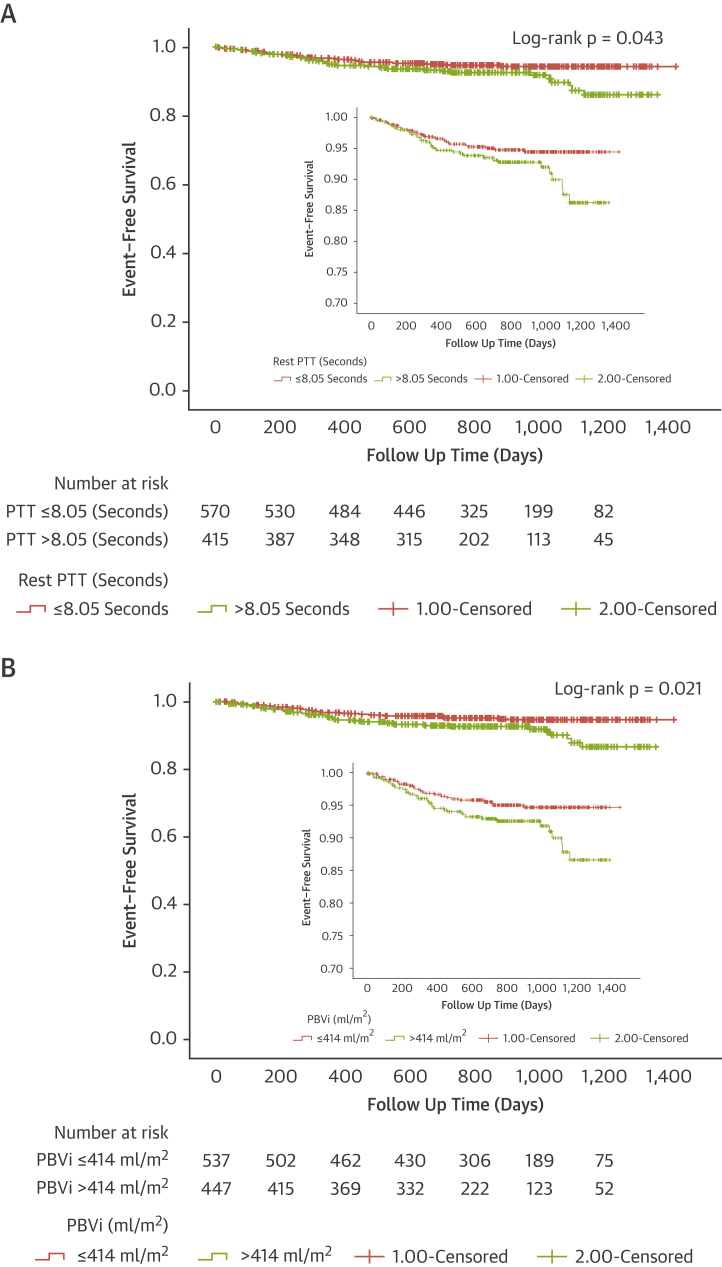
Kaplan-Meier Event-Free Survival Curves for Rest PTT and PBVi Event-free survival curves for major adverse cardiovascular events (heart failure hospitalization, myocardial infarction, stroke and ventricular tachycardia/implantable cardioverter-defibrillator treatment) according to mean PTT (8.05 s) **(A)** and mean PBVi (414 ml/m^2^) **(B)**. Longer PTT and higher PBVi were associated with higher rates of major adverse cardiovascular events (log-rank p = 0.043 and p = 0.021, respectively). Abbreviations as in Figure 1.

Data on MACE was available over a median period of 28.6 months (IQR: 22.6 to 35.7 months) during which period there were 71 (7.2%) MACE in 61 (6.2%) patients. These included 29 (2.9%) myocardial infarctions, 10 strokes (1%), 23 (2.3%) hospitalizations for heart failure, and 9 cases of ventricular tachycardia or appropriate ICD treatment (0.9%). Patients with MACE had longer rest PTT (8.4 s; IQR: 7.1 to 10.5 s vs. 7.6 s; IQR: 6.3 to 9.1 s; p = 0.005) and larger PBVi (430 ml/m^2^; IQR: 360 to 542 ml/m^2^ vs. 398 ml/m^2^; IQR: 333 to 472 ml/m^2^; p = 0.009). A similar difference was observed with rest PTTn (8.5 s; IQR: 7.6 to 9.8 s vs. 9.2 s; IQR: 8.0 to 10.8s; p = 0.003). Patients with MACE were also older, and more frequently had a history of diabetes, hypertension, previous revascularization, and stroke (Table 3). Kaplan-Meier event-free survival estimate curves for rest PTT and PBVi are presented in Figure 3.

**Table 3:** Comparison of Patients With MACE During Follow-Up (Median 28.6 Months) With Patients Without MACE

	No MACE (n = 924)	MACE (n = 61)	p Value
Demographics			
Age, yrs	62 (52–70)	65 (59–74)	0.008
Male	624 (67)	46 (75)	0.149
BSA, kg/m^2^	1.9 (1.9–2.1)	1.9 (1.75–2.0)	0.356
Comorbidities			
Diabetes	253 (27)	28 (46)	0.002
Hypertension	543 (59)	47 (77)	0.005
Dyslipidemia	446 (48)	33 (54)	0.378
Previous PCI/CABG	277 (24)	29 (48)	0.004
Atrial fibrillation	118 (12)	11 (18)	0.238
Stroke or TIA	50 (5)	8 (13)	0.013
Cancer	95 (10)	5 (8)	0.602
Previous stroke	50 (5)	8 (13)	0.013
Smoking history	312 (34)	25 (42)	0.250
CMR parameters			
LVEDVi, ml/m^2^	75 (64–90)	85 (66–116)	0.001
LVSVi, ml/m^2^	45 (39–52)	44 (39–52)	0.439
LVEF, %	63 (55–69)	58 (39–65)	0.001
LVMi, g/m^2^	56 (47–67)	64 (53–77)	0.002
LA area index, cm^2^/m^2^	11.7 (10–13.7)	13.1 (11.6–16.5)	<0.001
Any late gadolinium enhancement	371 (40)	45 (73)	<0.001
Resting heart rate, beats/min	68 (60–77)	67 (60–75)	0.537
Cardiac output, l/min	5.99 (5.07–7.22)	5.83 (4.75–6.95)	0.275
Stress MBF, ml/g/min	2.00 (1.60–2.48)	1.52 (1.08–1.87)	<0.001
MPR	2.43 (1.91–2.98)	1.87 (1.47–2.37)	<0.001
Rest PTT, s	7.6 (6.4–9.1)	8.4 (7.1–10.5)	0.005
Rest PTTn	8.5 (7.6–9.8)	9.2 (8.0–10.8)	0.003
PBVi, ml/m^2^	398 (333–472)	430 (360–542)	0.009

Values are median (interquartile range) or n (%).

BSA = body surface area; MACE = major adverse cardiac events (myocardial infarction, stroke, heart failure admission, and ventricular tachycardia or appropriate implantable cardioverter-defibrillator treatment [including implantable cardioverter-defibrillator shock and/or antitachycardia pacing]); PBVi = pulmonary blood volume index; PTTn = pulmonary transit time normalized for heart rate; TIA = transient ischemic attack; other abbreviations as in Table 1.

All-cause mortality data was available over a median of 31.4 months (IQR: 26.7 to 38.3 months), and during this period, 53 (5.4%) patients died. There was no statistically significant difference in rest PTT (7.7 s; IQR: 6.5 to 9.1 s vs. 7.6 s; IQR: 5.9 to 10.7 s; p = 0.851), rest PTTn (8.4 s; IQR: 7.64 to 9.81 s vs. 8.8 s; IQR: 7.22 to 11.1 s; p = 0.347) and PBVi (402 ml/m^2^; IQR: 337 to 474 ml/m^2^ vs. 393 ml/m^2^; IQR: 287 to 512 ml/m^2^; p = 0.526) between patients who survived and those who died during the follow-up period.

In a multivariable-adjusted Cox regression analysis, both rest PTT and PBVI were independent predictors of MACE (Table 4). The model was adjusted for age, sex, diabetes, and hypertension as well as prognostic imaging parameters (LVEF, presence of late gadolinium enhancement). The adjusted hazard ratio (HR) for 1 × SD (2.39-s) increase in rest PTT for MACE was 1.43 (95% CI: 1.10 to 1.85; p = 0.007). The adjusted HR for 1 × SD (118 ml/m^2^) increase in PBVi was 1.42 (95% CI: 1.13 to 1.78; p = 0.002). A sensitivity analysis across various models was performed, with inclusion of variables in the multivariable models limited to prevent overfitting. Both rest PTT and PBVi remained predictive of MACE in additional models with different variables, including MPR, left atrial area index, history of dyslipidemia, and history of previous myocardial infarction/PCI/CABG (Supplemental Tables 1 to 3). Rest PTT and PBVi were highly correlated (r = 0.63), and performed similarly in terms of predicting MACE (Supplemental Figure S3).

**Table 4:** Cox Proportional Hazard Models for Rest PTT and PBVi as Predictors of MACE and All-Cause Mortality

Predictors	MACE	All-Cause Mortality
Rest PTT, s		
Unadjusted		
Hazard ratio (95% CI) per 1 × SD increase	1.59 (1.31–1.92)	1.14 (0.90–1.46)
p value	<0.001	0.283
Adjusted		
Hazard ratio (95% CI) per 1 × SD increase	1.43 (1.10–1.85)	0.85 (0.62–1.16)
p value	0.007	0.313
Model chi-square value	53.79	79.14
Pulmonary blood volume index, ml/m^2^		
Unadjusted		
Hazard ratio (95% CI) per 1 × SD increase	1.46 (1.19–1.80)	0.98 (0.74–1.29)
p value	<0.001	0.872
Adjusted		
Hazard ratio (95% CI) per 1 × SD increase	1.42 (1.13–1.78)	0.95 (0.73–1.24)
p value	0.002	0.698
Model chi-square value	56.61	78.20

SD for PTT = 2.40 s; SD for PBVi = 118 ml/m^2^. Model for MACE was adjusted for age, sex, LVEF, diabetes, hypertension, and presence of LGE. Model for all-cause mortality was adjusted for age, LVEF, diabetes, hypertension, presence of LGE, and history of cancer. Both PTT and PBVi are independently associated with major adverse cardiovascular events but not all-cause mortality.

Abbreviations as in Table 1, Table 2 and Table 3.

In view of the possible interaction between early revascularization triggered by the perfusion CMR study itself and outcomes, we repeated the analysis after censoring cases undergoing early revascularization (defined as ≤90 days from perfusion CMR) (n = 17), with both rest PTT and PBVi remaining independently predictive of MACE. After adjusting for the same variables as those used in Table 4, the HR for 1 × SD increase in PTT for MACE was 1.33 (95% CI: 1.02 to 1.76; p = 0.038), and the adjusted HR for 1 × SD increase in PBVi was 1.379 (95% CI: 1.09 to 1.75; p = 0.007). Following normalization of PTT with heart rate (PTTn), the association with MACE and mortality remained unchanged. Additional sensitivity analysis of PTTn is shown in the Supplemental Table 4 and Supplemental Figure S2.

### Association of stress PTT with outcomes

PTT was also extracted during adenosine stress first pass perfusion, and an exploratory analysis between stress PTT and outcomes was performed. A total of 963 cases with stress PTT data were available for analysis, following exclusion of cases with incomplete or erroneous stress perfusion data. As expected, median stress PTT was shorter than rest PTT 6.2 s (IQR: 5.1 to 7.7 s) versus 7.7 s (IQR: 6.4 to 9.2 s), although they were highly correlated (r = 0.69; p < 0.001). Stress PTT also correlated with LVEF (r = −0.37), stress MBF (r = −0.31), LV end-diastolic volume index (r = 0.24), and LA area index (r = 0.32) (p < 0.001 for all). Over the follow-up period, 57 patients from this cohort had MACE. Stress PTT was predictive of MACE (p = 0.020) but not all-cause mortality (p = 0.064). The HR for 1-SD (2.64-s) increase in stress PTT was 1.34 (95% CI: 1.048 to 1.723; p = 0.020) after adjusting for age, LVEF, hypertension, diabetes, sex, and presence of LGE (Supplemental Table S5, Supplemental Figure S4).

## Discussion

This study investigated the prognostic power of pulmonary transit time and PBV measured automatically, in-line, during routine quantitative myocardial perfusion CMR. We demonstrate that PTT and PBVi are independently associated with adverse cardiovascular events in patients clinically referred for perfusion CMR, with a prognostic power incremental to established clinical risk factors and imaging biomarkers.

### PTT and blood volume as prognostic imaging biomarkers

PTT represents the average time it takes for a bolus of intravenous contrast to pass from the right to the left side of the heart (12). The potential clinical utility of PTT and the derived PBV has been the focus of extensive research for several decades (20). Invasive evaluation of PTT-derived PBV from right and left heart catheterization was shown to correlate with symptoms and New York Heart Association functional classification in patients with mitral stenosis (21) as well as in different models of heart failure and pulmonary hypertension (22).

Recently, a number of noninvasive imaging modalities, including echocardiography (23), computed tomography (CT) (8), and CMR (2,5,9,10), have been deployed to measure PTT, but clinical adoption and at-scale evaluation was hindered by the need for manual segmentation and data extraction. Kinetic analysis of the arterial input function curves derived from first-pass perfusion incorporates a combination of structural and hemodynamic parameters of the cardiopulmonary axis, providing a physiological framework supporting the association of PTT with adverse cardiovascular outcomes. The clinical endpoints tested in this study share common pathophysiological mechanisms, including macrovascular and microvascular alterations, changes in blood flow patterns (24), and endothelial dysfunction (25), processes that are likely to have a physiological impact on PTT and PBVi. As PTT and PBVi appear to serve as surrogate biomarkers of cardiopulmonary disease and are known to correlate with biochemical (brain natriuretic peptide), clinical (6-min walk test, New York Heart Association functional classification) and structural and hemodynamic parameters (cardiac output, pulmonary artery wedge pressure, valve disease severity), it is not surprising that these indexes are associated with conventional clinical endpoints. From a clinical perspective, PTT and PBVi potentially offer novel noninvasive imaging biomarkers that provide a more comprehensive assessment of cardiopulmonary physiology that do not focus on myocardial parameters in isolation (e.g., strain, LVEF, diastolic performance), but reflect a combination of physiological components, including chamber geometry, diastolic and systolic function, valve disease, as well as the pulmonary circulation.

Very few studies previously investigated the association of PTT parameters and outcomes, and these studies were focused on specific disease entities. During a median follow-up of 26 months (n = 112), Ricci et al. (2) showed that increased PBVi (>492 ml/m^2^) was independently associated with adverse outcomes in heart failure outpatients. Similarly, Swift et al. (5) showed that PTT was an independent predictor of mortality among 85 patients with pulmonary arterial hypertension over a 6-month follow-up. In our study, both PTT and PBVi were independently associated with MACE but not all-cause mortality. Data on the cause of death were not available for all patients, but the possibility of an association with cardiovascular mortality warrants further evaluation.

Data from previous CMR studies have shown PTT and PBVi to be increased in patients with impaired systolic LV function (2,3,9) and to be associated with markers of diastolic function in patients with hypertrophic cardiomyopathy (10). Using computed tomography data in patients with pulmonary hypertension, Colin et al. (8) recently demonstrated that PTT positively correlated with worsening degree of mitral regurgitation and increasing pulmonary artery wedge pressure estimates from right heart catheterization. In our study, PTT only moderately correlated with LVEF and LV end-diastolic volume index. However, compared with previous studies, the larger sample size and the broadly unselected patient population of patients with known or suspected coronary disease, including patients with variable pathologies (including valve disease, diastolic and systolic dysfunction, atrial fibrillation, and lung disease) might explain the slightly weaker correlation of PTT with cardiac parameters observed in our data.

A number of studies investigating the relation between PTT and cardiac volumes or biomarkers used a normalized PTT by adjusting for heart rate. The method of correction of PTT varied between studies (10,23), but given the association between heart rate and PTT also shown in our data (Figure 2, Table 2), we performed a further analysis using PTT normalized for heart rate (PTTn). PTTn was also predictive of MACE, and similarly to PTT and PBVi, was not predictive of all-cause mortality (Supplemental Table 4, Supplemental Figure S2). As the estimation of PBVi incorporates the use of cardiac output at rest, the impact of resting heart rate is incorporated in this metric.

Stress PTT extracted during adenosine stress perfusion was also found to be independently associated with outcomes. Stress PTT is, however, a very novel parameter, and lacks the previous invasive validation and clinical correlation work related to rest PTT. For example, the impact of adenosine, a pharmacological vasodilator stress agent, on the hemodynamic parameters that influence PTT during stress is not entirely clear, and may also differ between pharmacological agents and exercise. Stress PTT was also highly correlated with rest PTT in our cohort; therefore, the additive value of this parameter during stress over rest remains uncertain. Indeed, whether stress PTT performs differently in specific disease models warrants further research.

The present study exploits recent technical developments in perfusion CMR, allowing a fully automated process of analysis, making its adoption feasible within the clinical workflow setting. The measurement and reporting of PTT is done as part of a routine perfusion scan, and does not require additional planning or sequences (Central Illustration). Despite a small number of events, PTT and PBVi were shown to independently predict cardiovascular outcomes, with a predictive power incremental to well-established imaging biomarkers including LVEF and LGE, as well as more contemporary markers such as MPR (12). An exploratory analysis of stress PTT revealed a similar prognostic association with outcomes. The data presented highlight the need for systematic evaluation of PTT metrics in future prospective studies of selected disease cohorts, as these may provide additional insights into underlying pathophysiology and potential therapeutic targets.

**Central Illustration:**
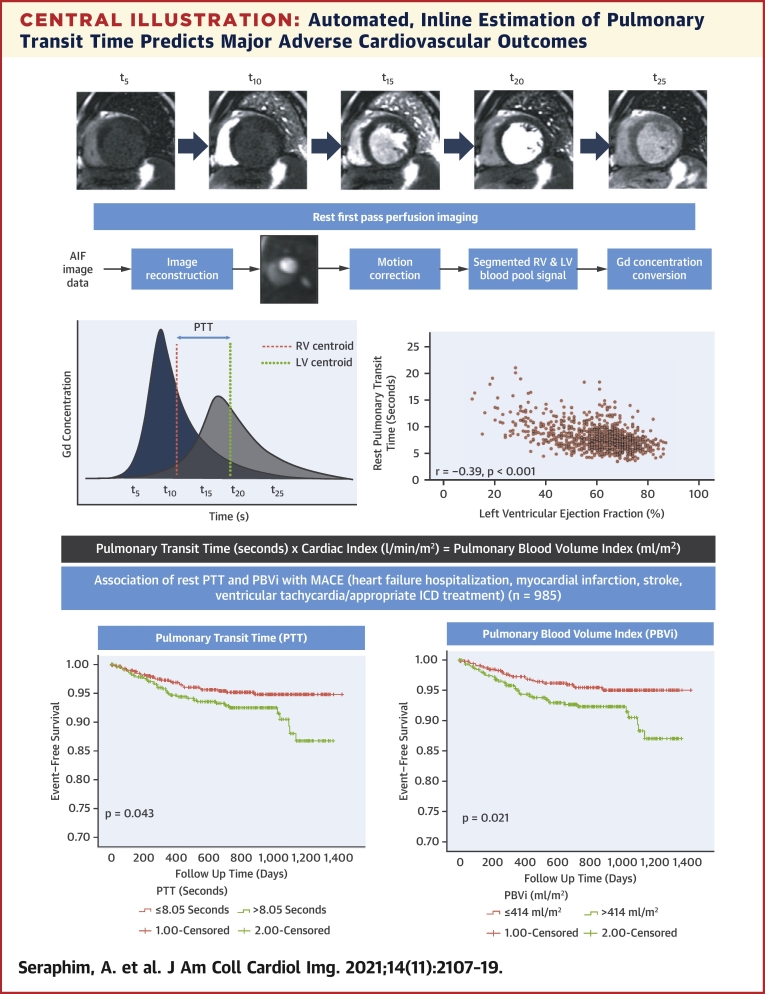
Automated, Inline Estimation of Pulmonary Transit Time Predicts Major Adverse Cardiovascular Outcomes Dynamic first-pass perfusion imaging of a basal short-axis slice showing the right ventricular (RV) and left ventricular (LV) cavities (t, seconds). Blood pool detection was performed automatically allowing estimation of gadolinium time-concentration curves in the RV and LV cavities. The **dashed lines** indicate the location of the centroid in each cavity, and the difference (i.e., the pulmonary transit time) between each centroid is indicated by the **arrow**. Kaplan-Meier curves (with log-rank tests) showing event-free survival for major adverse cardiovascular events.

### Study limitations

Despite adjusting for a number of clinical and CMR parameters, our analysis was not adjusted for indexes of diastolic dysfunction or valve disease (data for these only available in some cases), both of which are known to affect PTT. Stroke volume was calculated from planimetry of short-axis stack cine images rather than phase contrast velocity measurement, as the latter was not available. Although this may introduce a degree of error, particularly in the context of valve disease, this is not believed to alter the conclusion, as the PTT and PBVi varied over a much larger dynamic range than cardiac output. Furthermore, the study was designed primarily to assess the prognostic value of biomarkers (PTT and PBVi) that could be automatically derived from CMR sequences obtained as part of routine clinical imaging protocols. Although all first-pass perfusion studies rely on the indicator dilution principles, there are important variations between different methods of PTT estimation. Different sampling locations have been described, including the pulmonary trunk to left atrium (19), the RV to the left atrium, as well as the RV to LV (8,9,23). Evidently, the estimation of PTT and PBVi will vary depending on the anatomic landmarks selected. In our study, the RV and LV cavities were used for sampling as these can easily be sampled during the perfusion sequence, eliminating the need for additional planning and image acquisition. Patients had been clinically referred for myocardial perfusion CMR, and therefore the cohort predominantly included patients with known or suspected coronary artery disease. This may have introduced bias in terms of the association of PTT metrics. However, our analysis was adjusted for a number of cardiovascular risk factors as well as myocardial perfusion reserve, previously shown to independently predict adverse events within this patient cohort (12).

## Conclusions

PTT and PBV, measures of the cardiopulmonary system, can now be derived automatically without user input from latest-generation CMR perfusion mapping studies. Here, we show that these metrics are independently associated with adverse cardiovascular events over and above conventional factors, potentially providing clinically feasible imaging biomarkers of cardiopulmonary physiology.

Perspectives **COMPETENCY IN MEDICAL KNOWLEDGE:** PTT can be derived automatically from rest perfusion CMR imaging, without the need for additional image acquisition or user input. Both PTT and PBVi encode prognostic information which is independent of established imaging parameters including LVEF and the presence of LGE.

**TRANSLATIONAL OUTLOOK:** Further research is needed to establish whether PTT metrics can serve as noninvasive biomarkers for risk stratification and early warning signals in specific disease models and whether these can be altered with treatment.

## Funding Support and Author Disclosures

This study was supported by a Clinical Training Research Fellowship (to Dr Seraphim) from the British Heart Foundation (FS/18/83/34025) and directly and indirectly from the National Institute for Health Research Biomedical Research Centres at University College London Hospitals and Barts Health National Health Service Trusts. This study was also supported by the National Heart, Lung and Blood Institute, National Institutes of Health by the Division of Intramural Research (Z1A- HL006214-05 and Z1A- HL006242-02). This work forms part of the research areas contributing to the translational research portfolio of the Biomedical Research Centre at Barts, which is supported and funded by the National Institute for Health Research. Prof. Petersen has served as a consultant for and is a shareholder of Circle Cardiovascular Imaging Inc. All other authors have reported that they have no relationships relevant to the contents of this paper to disclose.
