# Supplementary material for: Prognostic Value of Pulmonary Transit Time and Pulmonary Blood Volume Estimation Using Myocardial Perfusion CMR
Source: JACC Cardiovasc Imaging. 2021 Nov;14(11):2107–19. doi: 10.1016/j.jcmg.2021.03.029 (PMC8560640; doi:10.1016/j.jcmg.2021.03.029)
Supplement: Supplemental Figures 1–4 and Supplemental Tables 1–5 [file mmc1.docx]

Data Supplement.


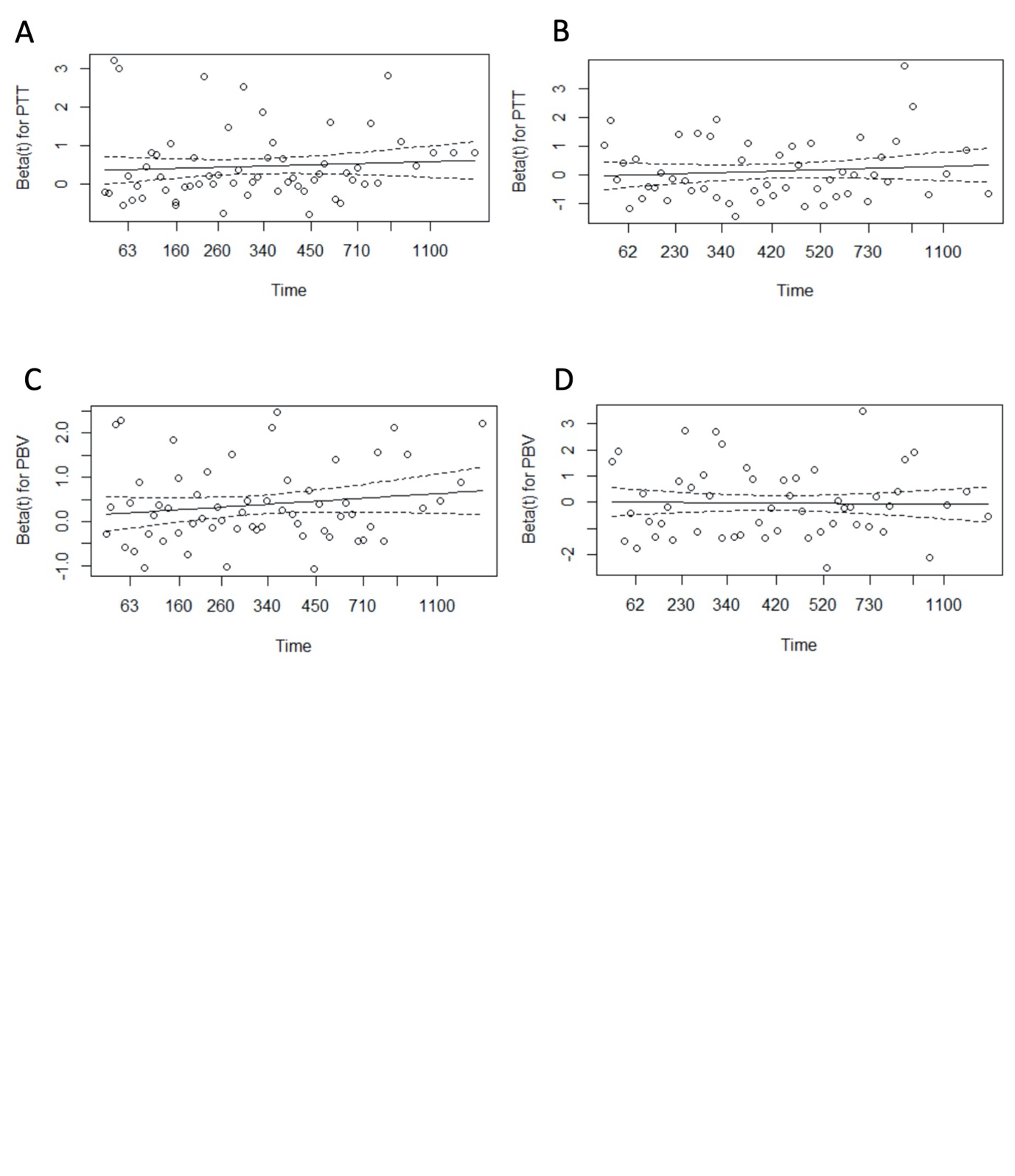


**Figure S1**. Graphs of scaled Schoenfeld residuals for (A) PTT with MACE (PH test p=0.49), (B) PTT with death (PH test p=0.43), (C) PBV with MACE (PH test p=0.18) and (D) PBV with death (PH test p=0.85)

| Table 1. Univariate Cox Regression analysis of associations with MACE | | | | |
| --- | --- | --- | --- | --- |
| **Predictors** |  | | **95% CI for HR** | |
| **Univariates** | P Value | Hazard Ratio (HR) | Lower Upper | |
| Rest PTT (seconds) | **<0.001** | 1.213 | 1.120 | 1.313 |
| Rest PTTn | **<0.001** | 1.162 | 1.091 | 1.238 |
| PBVi (ml/m^2^) | **<0.001** | 1.003 | 1.001 | 1.005 |
| Stress PTT (seconds) | **<0.001** | 1.174 | 1.100 | 1.254 |
| Cardiac output (L/min) | 0.359 | 1.000 | 1.000 | 1.000 |
| Cardiac index (CO/BSA) | 0.513 | 1.000 | 1.000 | 1.000 |
| Heart Rate (beats per minute) | 0.609 | 0.995 | 0.975 | 1.015 |
| LVEF (%) | **<0.001** | 0.966 | 0.951 | 0.982 |
| Age (years) | **0.002** | 1.035 | 1.012 | 1.059 |
| Diabetes | **0.003** | 2.146 | 1.297 | 3.511 |
| Hypertension | **0.008** | 2.249 | 1.238 | 4.086 |

| Table 2. Multivariable Cox Regression Analysis models of association between PTT and MACE | | | | | | | | |
| --- | --- | --- | --- | --- | --- | --- | --- | --- |
| **Predictors** |  | | | | | **95% CI for HR** | | |
| **Model 1 *Chi-square* value -45.38** | P Value | | Hazard Ratio (HR) | | | Lower Upper | | |
| PTT (seconds) | **0.003** | | 1.174 | | | 1.054 | 1.306 | |
| Age (years) | 0.217 | | 1.016 | | | 0.991 | 1.041 | |
| LVEF (%) | 0.133 | | 0.985 | | | 0.967 | 1.005 | |
| Diabetes | **0.007** | | 2.082 | | | 1.224 | 3.540 | |
| Hypertension | 0.117 | | 1.675 | | | 0.879 | 3.195 | |
| Dyslipidemia | 0.945 | | 0.982 | | | 0.584 | 1.651 | |
|  |  | |  | | |  |  | |
| **Model 2 *Chi-square* value -52.13** | P Value | | Hazard Ratio (HR) | | | Lower | Upper | |
| PTT (seconds) | **0.009** | | 1.151 | | | 1.035 | 1.281 | |
| Age (years) | 0.239 | | 1.015 | | | 0.990 | 1.039 | |
| LVEF (%) | 0.510 | | 0.993 | | | 0.974 | 1.013 | |
| LGE | **0.006** | | 2.539 | | | 1.310 | 4.921 | |
| Diabetes | **0.008** | | 2.051 | | | 1.204 | 3.491 | |
| History of MI, PCI and/or CABG | 0.731 | | 1.101 | | | 0.637 | 1.902 | |
|  |  | |  | | |  |  | |
| **Model 3 *Chi-square* value -52.33** | | *P* Value | | Hazard Ratio (HR) | Lower | | | Upper |
| PTT (seconds) | | **0.009** | | 1.156 | 1.036 | | | 1.005 |
| Age (years) | | 0.233 | | 1.015 | 0.991 | | | 1.040 |
| LVEF (%) | | 0.503 | | 0.993 | 0.974 | | | 1.013 |
| Diabetes | | **0.007** | | 2.071 | 1.221 | | | 3.514 |
| LGE | | **0.002** | | 2.636 | 1.418 | | | 4.902 |
| Atrial fibrillation | | 0.719 | | 0.882 | 0.446 | | | 1.747 |
|  |  | |  | | |  |  | |
| **Model 4 *Chi-square* value -66.35** | P Value | | Hazard Ratio (HR) | | | Lower | Upper | |
| PTT (seconds) | **0.028** | | 1.136 | | | 1.014 | 1.272 | |
| Age (years) | 0.534 | | 1.008 | | | 0.983 | 1.033 | |
| LVEF (%) | 0.452 | | 0.993 | | | 0.973 | 1.012 | |
| Diabetes | **0.019** | | 1.907 | | | 1.109 | 3.276 | |
| Myocardial perfusion reserve | **0.001** | | 0.520 | | | 0.348 | 0.778 | |
| LA area index (cm^2^/m^2^) | **0.041** | | 1.101 | | | 1.004 | 1.208 | |
|  |  | |  | | |  |  | |
| **Model 5 *Chi-square* value -65.06** | P Value | | Hazard Ratio (HR) | | | Lower | Upper | |
| PTT (seconds) | **0.004** | | 1.163 | | | 1.049 | 1.290 | |
| Age (years) | 0.481 | | 1.009 | | | 0.985 | 1.033 | |
| LVEF (%) | 0.709 | | 1.005 | | | 0.980 | 1.030 | |
| Diabetes | **0.036** | | 1.785 | | | 1.039 | 3.066 | |
| Myocardial perfusion reserve | **0.003** | | 0.535 | | | 0.355 | 0.805 | |
| LGE | **0.018** | | 2.162 | | | 1.141 | 4.096 | |
|  |  | |  | | |  |  | |
| **PTT = pulmonary transit time; PBVi = pulmonary blood volume index; LGE = Late gadolinium enhancement (incorporates both infarct and non-infarct pattern); LVEF = left ventricular ejection fraction; LA = left atrium; MPR = myocardial perfusion reserve** | | | | | | | | |

| Table 3. Multivariable Cox Regression Analysis models of association between PBVi and MACE | | | | |
| --- | --- | --- | --- | --- |
| **Predictors** |  | | **95% CI for HR** | |
| **Model 1 *Chi-square* value -47.79** | *P* Value | Hazard Ratio (HR) | Lower | Upper |
| PBVi (ml/m^2^) | **0.001** | 1.003 | 1.001 | 1.005 |
| Age (years) | 0.098 | 1.020 | 0.996 | 1.045 |
| LVEF (%) | **0.004** | 0.976 | 0.960 | 0.992 |
| Diabetes | **0.009** | 2.065 | 1.198 | 3.561 |
| Hypertension | 0.116 | 1.675 | 0.880 | 3.189 |
| Dyslipidemia | 0.955 | 0.985 | 0.586 | 1.656 |
|  | | | | |
| **Model 2 *Chi-square* value -51.18** | *P* Value | Hazard Ratio (HR) | Lower | Upper |
| PBVi (ml/m^2^) | **0.001** | 1.003 | 1.001 | 1.005 |
| Age (years) | 0.214 | 1.015 | 0.991 | 1.040 |
| LVEF (%) | 0.106 | 0.985 | 0.968 | 1.003 |
| Diabetes | **0.008** | 2.070 | 1.213§ | 3.535 |
| LGE | **0.008** | 2.463 | 1.264 | 4.799 |
| History of MI, PCI and/or CABG | 0.618 | 1.152 | 0.661 | 2.009 |
|  | | | | |
| **Model 3 *Chi-square* value -56.48** | *P* Value | Hazard Ratio (HR) | Lower | Upper |
| PBVi (ml/m^2^) | **0.002** | 1.003 | 1.001 | 1.005 |
| Age (years) | 0.230 | 1.015 | 0.991 | 1.040 |
| LVEF (%) | 0.096 | 0.985 | 0.968 | 1.003 |
| Diabetes | **0.015** | 2.070 | 1.213§ | 3.535 |
| LGE | **0.004** | 2.463 | 1.264 | 4.799 |
| Atrial fibrillation | 0.863 | 0.943 | 0.484 | 1.837 |
|  | | | | |
| **Model 4 *Chi-square* value -64.20** | *P* Value | Hazard Ratio (HR) | Lower | Upper |
| PBVi (ml/m^2^) | **0.024** | 1.003 | 1.001 | 1.005 |
| Age (years) | 0.312 | 1.012 | 0.988 | 1.037 |
| LVEF (%) | **0.045** | 0.983 | 0.966 | 1.000 |
| Diabetes | **0.018** | 1.935 | 1.122 | 3.340 |
| Myocardial perfusion reserve | **0.002** | 0.525 | 0.349 | 0.789 |
| LA area index (cm^2^/m^2^) | 0.103 | 1.085 | 0.984 | 1.196 |
|  |  |  |  |  |
| **Model 5 *Chi-square* value -65.53** | *P* Value | Hazard Ratio (HR) | Lower | Upper |
| PBVi (ml/m^2^) | **0.001** | 1.003 | 1.001 | 1.005 |
| Age (years) | 0.481 | 1.009 | 0.985 | 1.033 |
| LVEF (%) | 0.112 | 0.986 | 0.969 | 1.003 |
| Diabetes | **0.031** | 1.824 | 1.957 | 3.147 |
| Myocardial perfusion reserve | **0.003** | 0.539 | 0.356 | 0.814 |
| LGE | **0.018** | 2.164 | 1.141 | 4.103 |
|  |  |  |  |  |
| **PTT = pulmonary transit time; LGE = Late gadolinium enhancement (incorporates both infarct and non-infarct pattern); LVEF = left ventricular ejection fraction; LA = left atrium; MPR = myocardial perfusion reserve** | | | | |

| Table 4. Multivariate Cox Regression analysis of PTT normalised for heart rate (PTTn) and associations with MACE | | | | | |  |
| --- | --- | --- | --- | --- | --- | --- |
| **Predictors** |  | | | **95% CI for HR** | |  |
| **Model** **1** ***Chi-square* value –**  **53.43** | *P Value* | | Hazard Ratio (HR) | Lower | Upper |  |
| PTTn | **0.009** | | 1.134 | 1.031 | 1.247 |  |
| Age (years) | 0.308 | | 1.013 | 0.988 | 1.038 |  |
| Sex (male) | 0.995 | | 0.998 | 0.545 | 1.827 |  |
| LVEF (%) | 0.727 | | 0.727 | 0.976 | 1.017 |  |
| Diabetes | **0.022** | | 1.867 | 1.094 | 3.186 |  |
| Hypertension | 0.194 | | 1.517 | 0.809 | 2.846 |  |
| Presence of LGE | **0.001** | | 2.802 | 1.495 | 5.250 |  |
|  |  |  | |  |  | |
| **Model 2 *Chi-square* value -60.86** | *P* Value | Hazard Ratio (HR) | | Lower | Upper | |
| PTTn | **0.010** | 1.130 | | 1.030 | 1.240 | |
| Age (years) | 0.496 | 1.008 | | 0.984 | 1.033 | |
| LVEF (%) | 0.718 | 0.996 | | 0.976 | 1.017 | |
| Diabetes | **0.041** | 1.753 | | 1.022 | 3.005 | |
| Myocardial perfusion reserve | **0.005** | 0.560 | | 0.372 | 0.843 | |
| LGE | **0.005** | 2.460 | | 1.321 | 4.581 | |

**Figure S2**. Event-free survival curves for major adverse cardiovascular events (Heart failure hospitalization, myocardial infarction, stroke and ventricular tachycardia/ICD treatment) according to mean PTT normalised for heart rate (Mean PTTn=9.12). Log-rank p=0.003

**Figure S3**. Four-quadrant plot showing the distribution of cardiac events in 4 subgroups, separated by the cohort’s mean values (PTT= 8.05 seconds [horizontal line y-axis) and PBVi = 414ml/m^2^ [vertical line x-axis]).

*
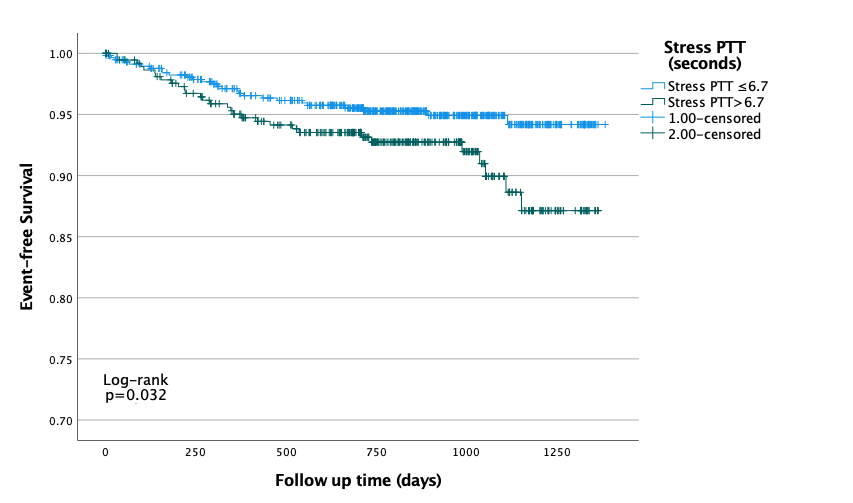
*

**Figure S4.** Event-free survival curves for major adverse cardiovascular events (Heart failure hospitalization, myocardial infarction, stroke and ventricular tachycardia/ICD treatment) according to mean stress PTT (Mean PTT=6.7). Log-rank p=0.032

| Table 5. Multivariate Cox Regression analysis of stress PTT and association with MACE | | | | | | |
| --- | --- | --- | --- | --- | --- | --- |
| **Predictors** |  | | **95% CI for HR** | | | |
| **Model** **1** ***Chi-square* value –**  **54.66** | *P Value* | Hazard Ratio (HR) | Lower | Upper | |  |
| Stress PTT(seconds) | **0.020** | 1.119 | 1.018 | | 1.230 | |
| Age (years) | 0.649 | 1.006 | 0.981 | | 1.032 | |
| Sex (male) | 0.296 | 0.722 | 0.392 | | 1.330 | |
| LVEF (%) | 0.384 | 0.991 | 0.971 | | 1.011 | |
| Diabetes | **0.061** | 1.708 | 0.976 | | 2.990 | |
| Hypertension | 0.107 | 1.732 | 0.889 | | 3.374 | |
| Presence of LGE | **<0.001** | 3.483 | 1.760 | | 6.893 | |
